# Beam selection for stereotactic ablative radiotherapy using Cyberknife with multileaf collimation

**DOI:** 10.1016/j.medengphy.2018.12.011

**Published:** 2019-02

**Authors:** James L. Bedford, Peter Ziegenhein, Simeon Nill, Uwe Oelfke

**Affiliations:** Joint Department of Physics, The Institute of Cancer Research and The Royal Marsden NHS Foundation Trust, London SM2 5PT, UK

**Keywords:** Cyberknife, Multileaf collimator, Beam-orientation selection, IMRT, Inverse planning, BOS, beam orientation selection, CT, computed tomography, IMRT, intensity-modulated radiotherapy, L-BFGS, Limited-memory Broyden–Fletcher–Goldfarb–Shanno, MLC, Multileaf collimator, PTV, Planning target volume, SABR, Stereotactic ablative body radiotherapy

## Abstract

The Cyberknife system (Accuray Inc., Sunnyvale, CA) enables radiotherapy using stereotactic ablative body radiotherapy (SABR) with a large number of non-coplanar beam orientations. Recently, a multileaf collimator has also been available to allow flexibility in field shaping. This work aims to evaluate the quality of treatment plans obtainable with the multileaf collimator. Specifically, the aim is to find a subset of beam orientations from a predetermined set of candidate directions, such that the treatment quality is maintained but the treatment time is reduced. An evolutionary algorithm is used to successively refine a randomly selected starting set of beam orientations. By using an efficient computational framework, clinically useful solutions can be found in several hours. It is found that 15 beam orientations are able to provide treatment quality which approaches that of the candidate beam set of 110 beam orientations, but with approximately half of the estimated treatment time. Choice of an efficient subset of beam orientations offers the possibility to improve the patient experience and maximise the number of patients treated.

## Highlights

• A fast optimisation framework is used to create IMRT SABR plans for Cyberknife.
• The value of the Cyberknife multileaf collimator is investigated.
• A beam selection algorithm is used to determine a subset of beam orientations.
• Fifteen selected beams are sufficient to create high-quality treatment plans.
• Treatment time is minimised using this approach.

## 1 Introduction

The Cyberknife system (Accuray Inc., Sunnyvale, CA) includes a multileaf collimator (MLC), which allows maximal flexibility in field shaping and fewer monitor units in stereotactic radiosurgery than with a cone collimator [1], [2]. The MLC consists of 26 leaf pairs, each of width 3.85 mm, giving a maximum field size of 115 mm × 100 mm at a nominal source-axis distance of 800 mm.

The standard beam set for a Cyberknife stereotactic ablative body radiotherapy (SABR) treatment uses 110 beams, referred to as nodes. These are typically non-isocentric and non-coplanar, and are chosen so as to provide a collision-free path for the delivery robot around the patient [1]. However, such a large number of beams is unlikely to be necessary for many, if not all, treatment sites, and may lead to an excessive treatment delivery time without much benefit [3]. This work therefore aims to determine an optimal subset of beams for each patient, such that the treatment quality approaches that of the full node set. This is accomplished firstly by examining predetermined beam subsets defined by the manufacturer, and secondly by applying a beam selection technique.

A number of approaches have previously been used for beam orientation selection in radiotherapy. As well as the implementation of methods for conformal radiotherapy [4], the more complex problem of determining beam orientations and fluence maps for intensity-modulated radiotherapy (IMRT) has been approached by beam's eye view score methods [5], [6], combination of individually selected beams [7], successive addition of beams to a pool [8], [9], [10], angle perturbation [11], [12], [13] and cluster analysis [14]. Other methods have also been reported [15], [16], [17], [18], [19], [20], [21]. All of these methods benefit from fast optimisation methods [22], [23] and comparisons of methods have helped to clarify the benefits of these approaches [24], [25].

Some of the recent work on trajectory optimisation for arc therapy can also be applied usefully to the question of beam orientation selection for Cyberknife. For example, Smyth et al. [26], [27] find the least cost path through a cost function map based on individual beam metrics. Wild et al. [28] also use a path connection algorithm to find the shortest path between desirable orientations. Locke and Bush [29] also use a path search algorithm, but take into account the connectedness of the areas of the beam's eye view which are useful for beam delivery.

Several methods have focused specifically on the Cyberknife device. For example, Kearney et al. [30] describe a method for producing arc trajectories for Cyberknife. A subset of optimal beams is selected from a complete library of beams, and then these beams are joined using a path selection method, and formed into a continuous arc.

## 2 Methods and materials

### 2.1 Patients and treatment plans

Four patient cases were considered in this study, with tumour sites of prostate and base of seminal vesicles, lung, liver and partial breast. The prostate case was planned with two distinct techniques, as described below, leading to a total of five types of treatment plan. All treatment plans used a SABR technique, with hypofractionated dose prescriptions of 3–5 fractions (see Table 1).

**Table 1:** Fractionation schemes used in this study.

Case	Total Dose (Gy)	Fractions	Protocol
Prostate A	36.25	5	RTOG 0938
Prostate B	38.00	4	Fuller et al. [31], [32]
Lung	50.00	5	RTOG 0813
Liver	42.75	3	Vautravers–Dewas et al. [33]
Partial breast	35.00	5	RTOG 0413

**Fig. 1:**
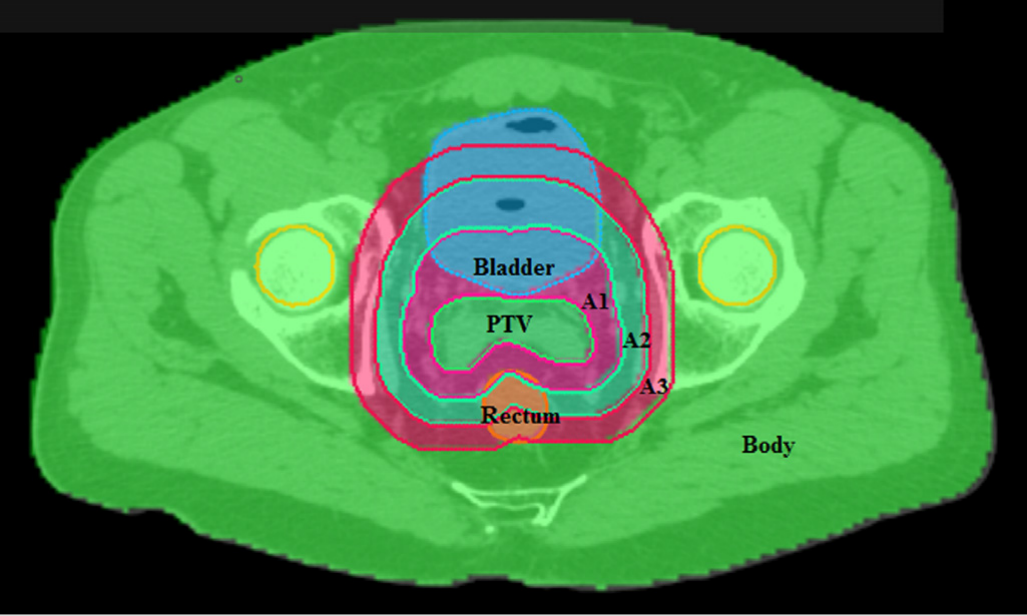
Overlap and priorities. Planning target volume (PTV) has the highest priority, followed by critical structures such as rectum and bladder. Three annular structures, (A1, A2, A3) with width 10 mm then follow, and the remainder of the body then has the lowest priority.

Patient cases were imported into the in-house treatment planning system DynaPlan and dose was calculated using a standalone dose calculation module supplied by Accuray Inc., so as to accurately represent dose delivered by the Cyberknife system. The computational framework required that appropriate priorities were assigned to the different anatomical structures outlined on the CT images so that the optimizer would work correctly in the case of overlap (see Fig. 1). Each voxel in the volume was assigned to one structure only.

**Fig. 2:**
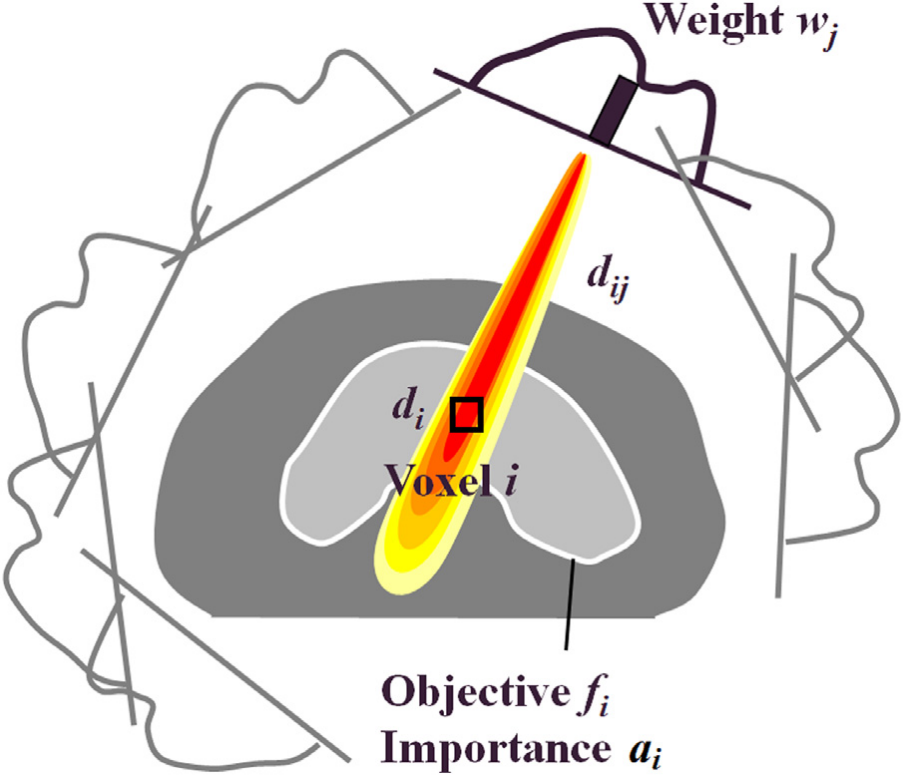
Dose model.

The inverse planning method required the dose, *d_i_*, at voxel *i* to be calculated as [28]:

(1) $$d_{i}=\sum_{j}d_{ij}w_{j},$$

where *d_ij_* was the dose at voxel *i* due to fluence *w_j_* at element *j* of the intensity matrices (Fig. 2).

Accordingly, the dose-influence matrix *d_ij_* was determined by calculating dose distributions for fields of one intensity bixel in size. This was an approximation as the dose due to a single large field was not exactly equal to the sum of doses delivered by a sum of individual bixels, but was considered accurate enough for this study. In all cases, the fluence bixel size was 2 × MLC leaf width by 5 mm and the fluence grid approximately covered the beam's eye view of the PTV with a 5 mm margin. In some regions of some of the beams, the fluence grid was greater in extent than the PTV, and in others, it was less. This imperfection was not found to have a significant impact on the results. The calculation voxel size was 2 × CT pixel width by 2 × CT pixel height × CT slice spacing. A lower dose threshold of 0.015% of the maximum dose of each *d_ij_* component was used, which in practice meant that all scattered dose was incorporated into the inverse planning. The *d_ij_* matrices covered the entire patient, so that the components relating to each beam totalled approximately 1 GB in size. All dose voxels were used in structures for which optimisation objectives were specified.

Each treatment plan consisted of 110 beam orientations, using an average of two apertures per beam orientation (node). Treatment plans were optimized using an objective function, *F*, summed over a number of volumes, *i,* each with individual objective value *f_i_*:

(2) $$F=\sum_{i}f_{i},$$

with *f_i_* defined as:

(3) $$f_{i}=a_{i}{\left[d_{i}^{\min}-d_{i}\right]}_{\geq 0}^{2}+a_{i}{\left[d_{i}-d_{i}^{\max}\right]}_{\geq 0}^{2}$$

where *a_i_* was a structure-specific importance factor. Both the minimum and maximum terms were used for targets, while only the maximum term was used for normal tissues. A number of iterations, *x*, of an iterative gradient descent method were then used to reach a solution for the intensity values in the fluence matrix:

(4) $$w_{{}_{j}}^{x+1}={\left[w_{j}^{x}-\alpha p_{j}^{x}\right]}_{\geq 0}.$$

where α was a relaxation parameter. The direction vector *p^x^* was *in principle* given as:

(5) $$p^{x}={\left[\nabla^{2}F\left(w^{x}\right)\right]}^{-1}\nabla F\left(w^{x}\right).$$

However, the low-memory Broyden–Fletcher–Goldfarb–Shanno (L-BFGS) method was used to avoid the memory-intensive calculation of the inverse Hessian matrix [∇^2^*F*(*w^x^*)]^ − 1^. In this scheme, the direction vectors were obtained by a recursion relation [28]:

(6) $$p^{x+1}=p^{x}+B\left(F,\nabla F\right).$$

Following fluence optimisation, sequencing was carried out using a standard sequencing method [34], and aperture optimisation was then carried out, also using a gradient descent method [28], [35]. This method converted the aperture optimisation problem into a fluence optimisation problem, so that the same L-BFGS method could be used for aperture optimisation as for fluence optimisation. No attempt was made to optimise the numbers of beam directions, apertures or monitor units (MU) in the final plan.

**Fig. 3:**
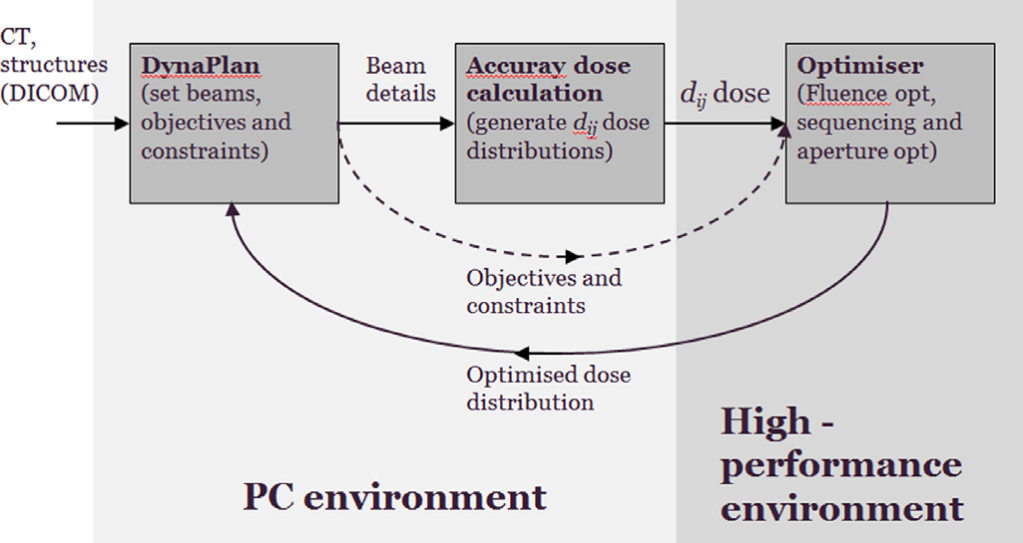
Computational system.

This method was implemented in a fast multi-threaded planning framework [35]. This enabled a solution for 110 nodes to be obtained in less than 15 min for 40 fluence iterations and 40 iterations of direct aperture optimisation (see Fig. 3). The optimisation itself was implemented in a high-performance environment, which was a dual Intel Xeon E5-2650 with 128 GB RAM.

**Fig. 4:**
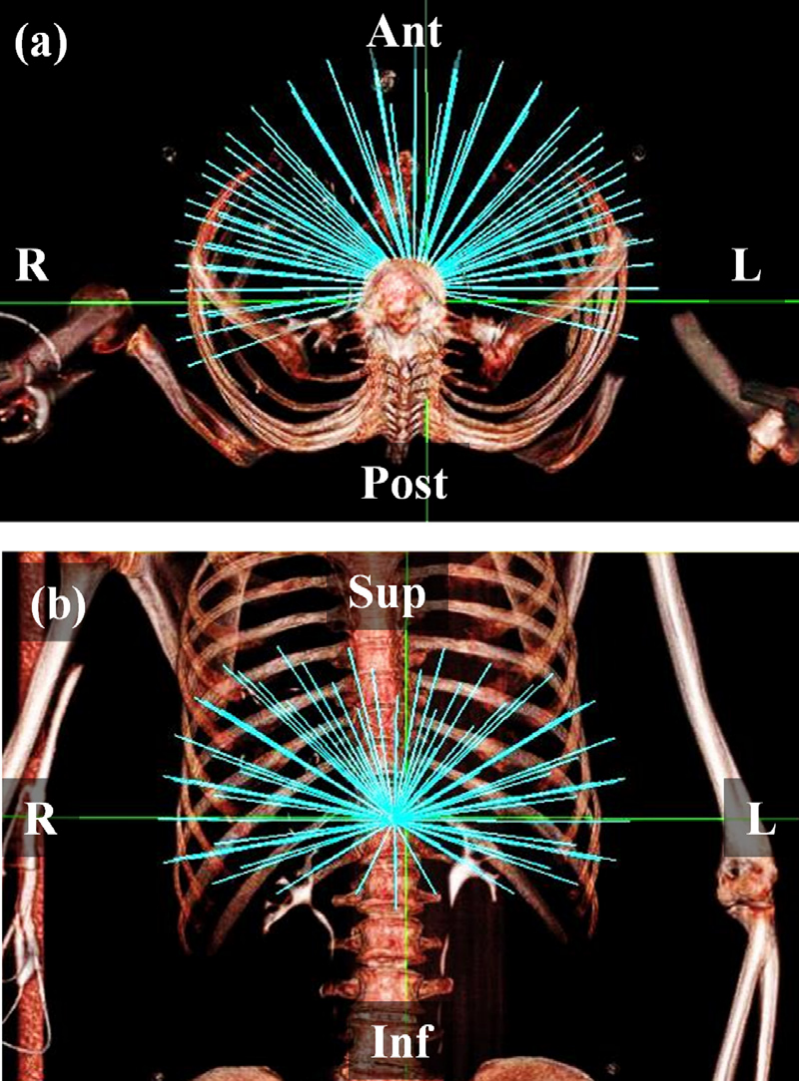
The body nodeset from which the beams were selected. (a) transaxial view, (b) coronal view.

The starting point for comparisons was to use an Accuray-supplied body nodeset with 110 nodes (Fig. 4), with a variable number of apertures being specified to the optimizer. This number was 2 for the prostate cases, 1 for liver and breast cases, and 3 for the lung case, reflecting the degree of intricacy required in the solution. An Accuray-supplied subset of the body nodeset, containing 36 nodes spaced evenly over the same total solid angle, was also used. This approach was similar to the standardised bouquet determined by Yuan et al. [36].

**Fig. 5:**
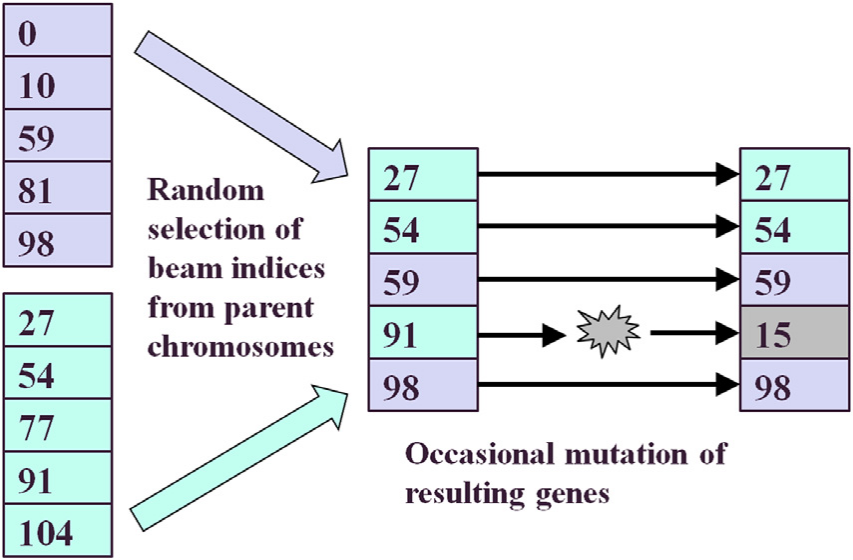
Concepts involved in the evolutionary algorithm used in this work. The two left-hand lists represent two node sets in a population, the numbers representing node indices. The offspring has features of both of these two individuals, with occasional mutations.

Beam selection was carried out using a variation of the evolutionary algorithm of Li et al. [37], which was also similar to the approach of Hou et al. [38], who used an evolutionary algorithm for orientation selection and a simulated annealing algorithm for intensity calculation. The concept of nesting an intensity calculation inside a beam orientation loop was also used by Rowbottom et al. [39]. Using this method, 15 beams were selected from the 110-node body nodeset. Other numbers of beams were investigated and 15 beams were found to be the practical minimum that allowed for production of a high-quality dose distribution. For all orientation-selected cases, five segments per beam were allowed, except for the liver case, where the relative simplicity of the planning target volume (PTV) required only three segments per beam to be used. The method is summarised in Fig. 5.

A population of 20 plans was used in this work, representing a collection of plans whose properties were to be successively improved by the evolutionary algorithm. The beam orientations for each plan were chosen initially by randomly selecting beam indices from the candidate node set of 110 beams. This population of individual treatment plans, or individuals, then underwent 20 iterations, or generations. The generation was defined as the population at a given phase in the optimisation process. At each iteration, 20 new individuals were generated from the current 20 individuals, to form the next generation. In this way, the population was maintained at 20 throughout the scheme. The genetic encoding consisted of a list of beam indices used by each individual or treatment plan. Note that the fitness function was taken to be the objective function, *F*, as defined in Eq. (2), with lower values representing greater fitness.

To generate a new individual, the fittest tenth of the current population was identified according to objective function value, and these two individuals were combined. Each gene, i.e. each beam index, of the new individual was determined by randomly using a beam index from either of the parent genes. In this crossover or recombination operation, the probability of using a beam index from one parent was 0.4 and the probability of using a beam index from the other was 0.6, following empirical tests. If the new beam index was identical to a beam index already existing in the new individual, another attempt was made to generate that particular beam index, and if this also matched an existing beam index, it was accepted anyway. This new beam index then underwent mutation, with a probability of 0.05. This involved replacing it with another beam index from the set of candidate beam indices. As there was no simple relationship between beam index and beam orientation, (i.e. beams with adjacent indices did not necessarily have adjacent beam orientations), no attempt was made to select similar indices or orientations. Mutation therefore involved a change of index and orientation that could be considerable.

The end result of this process was that each randomly selected pair of individuals gave rise to an offspring. After 20 of such offspring were generated, they replaced the original individuals, so that a new generation of 20 plans was produced. Each of these plans was then optimised using 20 fluence iterations, sequencing, and 20 iterations of direct aperture optimisation. The whole process was then repeated for 20 iterations. In the implementation of Li et al. [37], the optimal plan was taken as the fittest individual in the final generation. However, in our implementation, the optimal plan was taken as the fittest individual to be found in any of the generations. This was used to provide a similar effect to elitism, in which the fittest individuals are retained for subsequent generations.

The parameters described above were chosen following empirical tests to determine the optimum settings. To demonstrate that the selected values were optimum, the values were perturbed and the progress of the beam orientation selection (BOS) was evaluated for the prostate B case and the liver case. Several different combinations of the crossover proportions and the mutation rate were evaluated. Furthermore, to evaluate the statistical accuracy of the evolutionary algorithm, these cases were recalculated 25 times using different seed values in the random number generator. The adequacy of the number of plans and number of generations was also assessed for the prostate B case by recalculating using 100 plans in 100 generations.

Sometimes the importance factors were adjusted during production of the plans using BOS. This was mainly to reduce the surface dose when using relatively few beam orientations. This meant that the objective values were different for the BOS plan and the reference plan produced from the body or even path, even for identical dose distributions. Consequently, where objective values were compared, the BOS objective value was compared against that for a re-optimised plan using the body or preset short path but with the same importance factors as used in the BOS plan.

Plans were compared using dose statistics and conformity index, which was defined as the volume receiving the prescribed dose divided by the volume of the planning target volume. Treatment times were estimated according to a vendor-supplied algorithm incorporating initial patient setup, beam-on, MLC reshaping between apertures, robot traversal between nodes and imaging.

## 3 Results

**Fig. 6:**
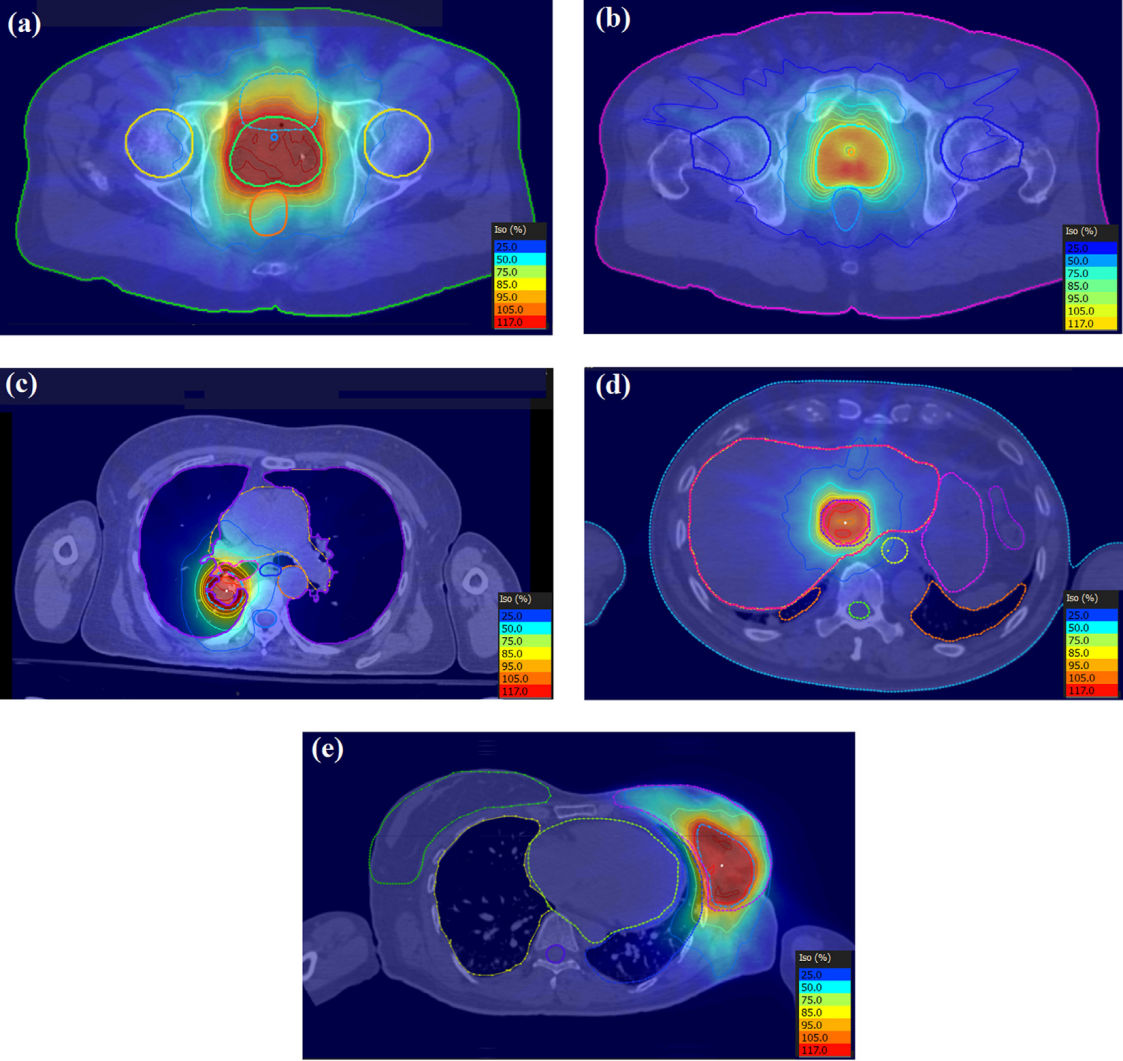
Transaxial dose distributions for (a) prostate A, (b) prostate B, (c) lung, (d) liver and (e) partial breast cases. The dose levels as a percentage of the prescription dose are shown in each case.

The transaxial dose distributions are shown in Fig. 6 for the five cases with the 110-node body node set. The dose distributions shown are for the *d_ij_*-based dose distribution output from the optimiser, without recalculation of the apertures as complete beams. It can be seen that the dose distributions are conformal in nature, with appropriate sparing of organs at risk near to the PTV. A summary of results over the five patients for the body path, the preset short path and BOS path are shown in Table 2.

**Table 2:** Plan statistics for the five patient cases.

	Body path	Preset short path	Orientation selected path
Number of nodes	110	36	15
Inverse planning time per run (mins)	10	5	120
Median apertures	310	158	165
Median MU per Gy	1575	1881	1499
Median conformality index	1.03	1.05	1.06
Median estimated treatment time (mins)	51	37	34

The orientation selection takes longer for inverse planning than the other techniques due to the number of plan optimisations required. The number of apertures approximately follows the number of nodes in the plan for the body path and the preset short path, although there is a similar number of apertures for the BOS path as with the preset short path, for rather fewer beams. The estimated treatment time follows accordingly. The monitor units per gray and conformity index are approximately constant for all three of the types of treatment plan.

**Fig. 7:**
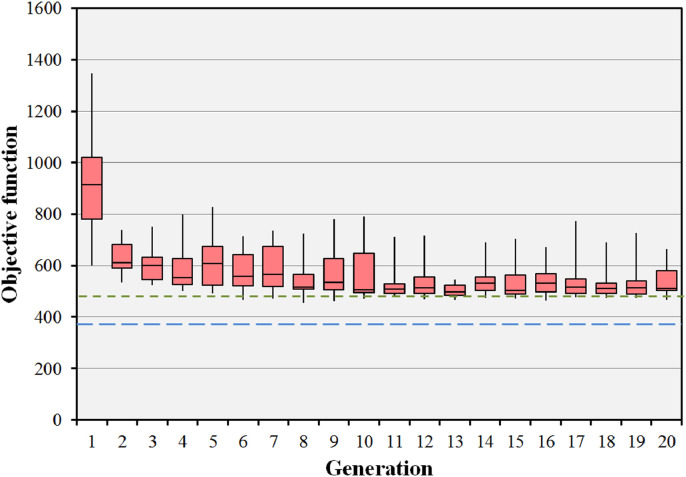
Convergence of the objective function for the prostate B case with 15 beams selected from 110 candidate beams. The boxes represent the median and the 25th and 75th percentiles of the 20 individual objective function values at each generation. The error bars represent the range of these 20 objective values. The green dotted line shows the objective value for the preset short path and the blue dashed line shows the objective value for the body path. (For interpretation of the references to colour in this figure legend, the reader is referred to the web version of this article.)

**Fig. 8:**
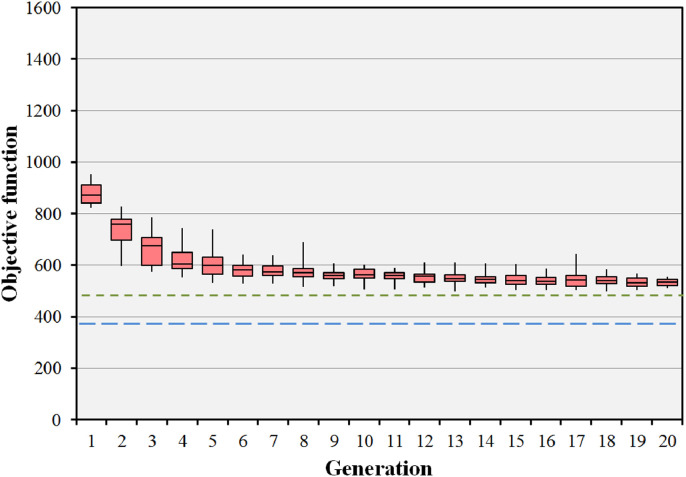
Statistical performance of the evolutionary algorithm for the prostate B case. The algorithm has been run 25 times and the median objective value of the population of 20 individuals recorded for each run. The boxes represent the median and the 25th and 75th percentiles of the 25 median objective function values at each generation. The error bars represent the range of these 25 median objective values. The green dotted line shows the objective value for the preset short path and the blue dashed line shows the objective value for the body path. (For interpretation of the references to colour in this figure legend, the reader is referred to the web version of this article.)

The impact of the parameters used in the evolutionary algorithm on the median final objective value for the BOS result in the prostate B case is shown in Table 3. Run 1, the standard case, is shown to be competitive with the other runs using different parameters. Only run 7 has a median final objective value which is appreciably lower than that of run 1, but takes many hours to achieve the result. Fig. 7 shows the objective values for 20 iterations of the BOS scheme for the prostate B case, corresponding to run 1 of Table 3. The optimisation rapidly reaches convergence to a fit population of treatment plans. The minimum objective value encountered is better than that of the preset short path and approaching that of the body path. It can be seen that there is scope to reduce the number of iterations, as the global solution is found relatively rapidly. Fig. 8 shows the results of the evolutionary algorithm for the same case when the BOS scheme is repeated 25 times (run 6). The same pattern of convergence is seen as with the single run, and the small range of the median objective function values shows that the algorithm is statistically stable. Note that Fig. 7 shows the objective values of the individuals, whereas Fig. 8 shows the median objective values of various runs.

**Table 3:** Impact of varying the parameters of the evolutionary algorithm in the prostate B case. The pertinent changes in parameter values are shown in bold type. Note that the median and range final objective values for run 6 relate to the median value attained by the population at each of multiple runs rather than the value attained by the individuals at a single run.

Parameter	Run 1	Run 2	Run 3	Run 4	Run 5	Run 6	Run 7
Population size (plans)	20	20	20	20	20	20	**100**
Generations	20	20	20	20	20	20	**100**
Statistical repeats	1	1	1	1	1	**25**	1
Crossover ratio	0.4	**0.3**	**0.5**	0.4	0.4	0.4	0.4
Mutation probability	0.05	0.05	0.05	**0.01**	**0.1**	0.05	0.05
Final objective median	512	509	515	518	526	536	493
Final objective range	466–664	483–659	461–568	482–594	489–849	512–555	432–945
Lowest objective found	456	481	457	482	485	447	418

**Fig. 9:**
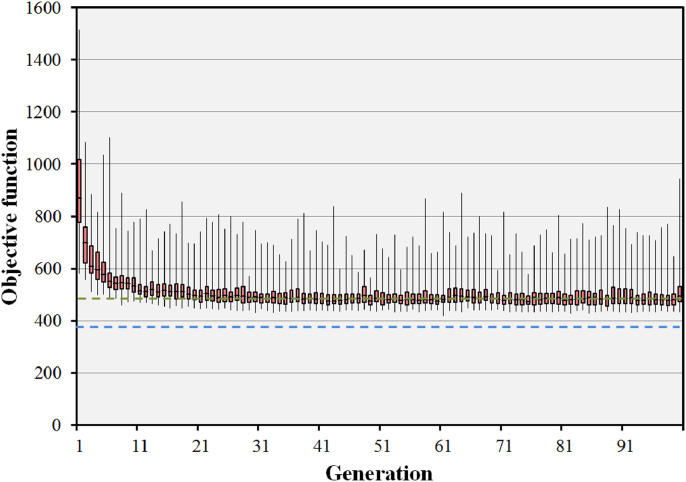
Convergence of the objective function for the prostate B case with 15 beams selected from 110 candidate beams. In this run, 100 individuals in 100 generations are used. The boxes represent the median and the 25th and 75th percentiles of the 100 individual objective function values at each generation. The error bars represent the range of these 100 objective values. The green dotted line shows the objective value for the preset short path and the blue dashed line shows the objective value for the body path. (For interpretation of the references to colour in this figure legend, the reader is referred to the web version of this article.)

Similar results are also seen in Fig. 9 for the case of 100 individuals in 100 generations (run 7). Although there is a large range in the objective function values at each generation, the median objective value reaches a constant value after around 20 iterations. The objective value reaches a slightly smaller value than in Fig. 7, showing that there is a small additional benefit in the larger population size. However, the benefit is not large, and the use of 20 individuals in 20 generations is considered to be adequate for the purposes of providing good quality dose distributions.

**Fig. 10:**
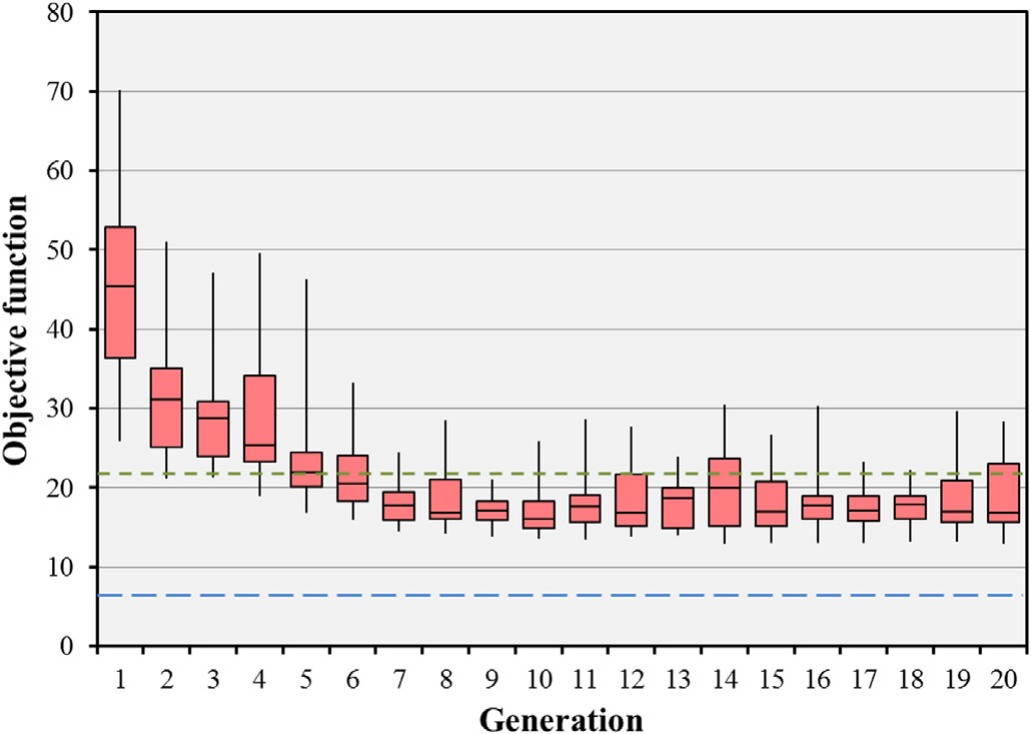
Convergence of the objective function for the liver case with 15 beams selected from 110 candidate beams. The boxes represent the median and the 25th and 75th percentiles of the 20 individual objective function values at each generation. The error bars represent the range of these 20 objective values. The green dotted line shows the objective value for the preset short path and the blue dashed line shows the objective value for the body path. (For interpretation of the references to colour in this figure legend, the reader is referred to the web version of this article.)

**Fig. 11:**
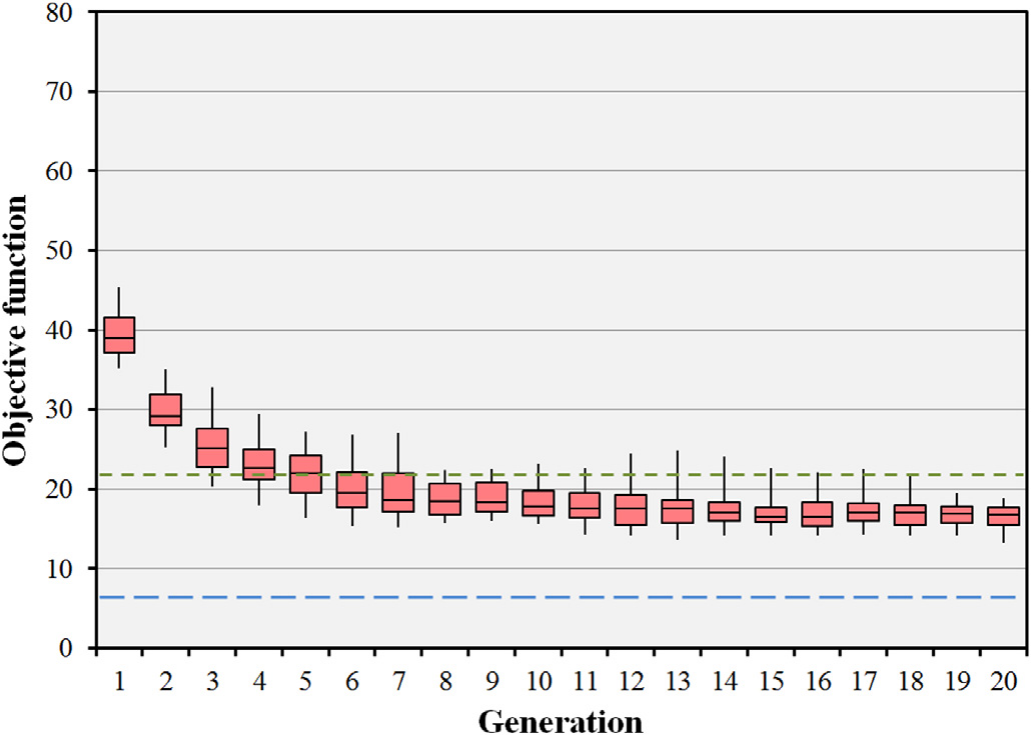
Statistical performance of the evolutionary algorithm for the liver case. The algorithm has been run 25 times and the median objective value of the population of 20 individuals recorded for each run. The boxes represent the median and the 25th and 75th percentiles of the 25 median objective function values at each generation. The error bars represent the range of these 25 median objective values. The green dotted line shows the objective value for the preset short path and the blue dashed line shows the objective value for the body path. (For interpretation of the references to colour in this figure legend, the reader is referred to the web version of this article.)

The impact of the parameters used in the evolutionary algorithm on the median final objective value for the BOS result in the liver case is shown in Table 4. As with the prostate B case, the standard run (run 1), is shown to produce final objective values which are competitive with the other runs. The difference in magnitude of the objective function values, compared to the prostate B case, is a reflection of the different anatomical structures and importance factors used for the two cases, and comparison of these values between the cases is therefore not meaningful. Fig. 10 shows the objective values for 20 iterations of the BOS scheme for the liver case, corresponding to run 1 of Table 4. The optimisation rapidly reaches convergence to a fit population of treatment plans. The final objective value is better than that of the preset short path and approaching that of the body path. Fig. 11 shows the results of the evolutionary algorithm for the same case when the BOS scheme is repeated 25 times (run 6). Again, the small range of the median objective function values shows that the algorithm is statistically stable.

**Table 4:** Impact of varying the parameters of the evolutionary algorithm in the liver case. The pertinent changes in parameter values are shown in bold type. Note that the median and range final objective values for run 6 relate to the median value attained by the population at each of multiple runs rather than the value attained by the individuals at a single run.

Parameter	Run 1	Run 2	Run 3	Run 4	Run 5	Run 6
Population size (plans)	20	20	20	20	20	20
Generations	20	20	20	20	20	20
Statistical repeats	1	1	1	1	1	**25**
Crossover ratio	0.4	**0.3**	**0.5**	0.4	0.4	0.4
Mutation probability	0.05	0.05	0.05	**0.01**	**0.1**	0.05
Final objective median	16.8	17.7	19.3	15.1	18.9	16.7
Final objective range	12.9–28.3	14.6–28.2	14.0–25.8	13.1–18.0	14.7–27.1	13.3–18.8
Lowest objective found	12.9	14.0	14.0	13.0	11.9	11.0

In terms of the clinical quality of the treatment plans, the clinical constraints are met in most cases where a solution is feasible. There are several cases, notably prostate PTV overlapping the rectum, and lung PTV overlapping with the proximal bronchial tree, where a solution is infeasible for certain beam arrangements. In addition, there are several other instances where the constraints are not met due to the PTV overlapping with a critical structure, which is difficult to handle due to the requirement in the computational framework to have only one structure defined at each location (see Fig. 1). The PTV is always set to the highest priority, so it is difficult to control the dose in the regions where a critical structure overlaps with the PTV. However, in general, clinical constraints are met in the cases presented.

**Fig. 12:**
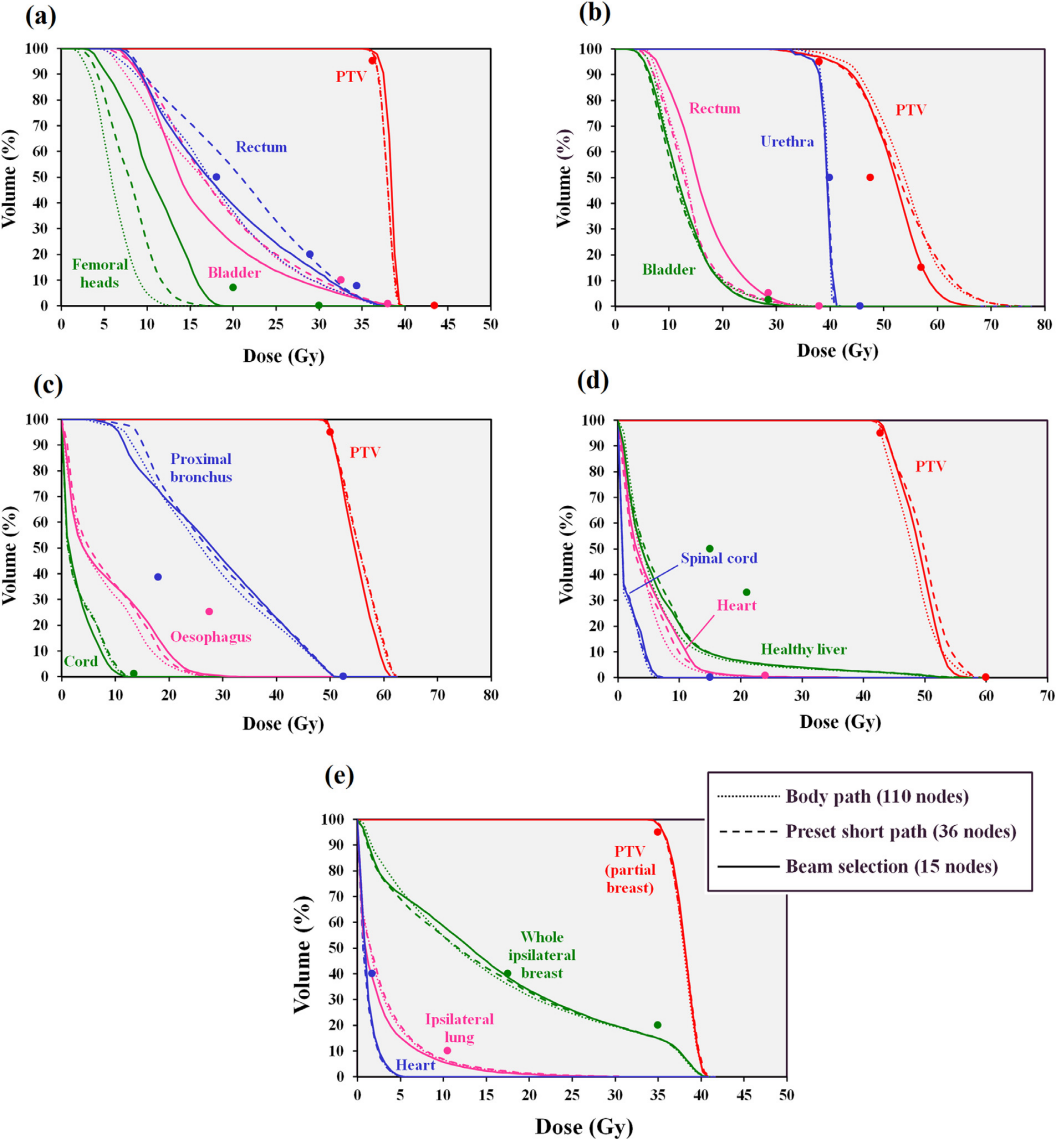
Dose-volume histograms for (a) prostate A, (b) prostate B, (c) lung, (d) liver and (e) partial breast cases. Dotted lines: body path (110 nodes), dashed lines: preset short path (36 nodes), solid lines: BOS path (15 nodes). The principal dose constraints are shown as points. The PTV constraints in (b) are all minimum dose constraints.

Dose-volume histograms comparing the body path, the preset short path and the BOS path are shown in Fig. 12. For the prostate A case, the PTV receives a similar dose with the body path, the preset short path and the BOS path. The rectal dose passes the 18.12 Gy at 50% volume constraint with the full path and the BOS path but fails with the preset short path. Meanwhile the bladder dose is lower with BOS than with the body path and the femoral head dose is higher than with the body path. However, these doses are within tolerance (principally 18.12 Gy at 50% for the bladder and 20 Gy at 10 cm^3^ for the femoral heads) for all of the techniques.

For the prostate B case, the PTV dose for the preset short path is similar to that for the body path, while the dose with BOS is slightly less, which in the context of this brachtherapy-like boost protocol, represents a reduction in plan quality. However, all techniques give PTV dose which meets the constraints (at least 38.00 Gy at 95%, at least 47.50 Gy at 50% and at least 57.00 Gy at 15%). The urethra dose is similar with all techniques and within the tolerance of 39.90 Gy at 50%. Rectum dose is slightly higher with BOS than with the body path or the preset short path, but again within tolerance (principally 28.50 Gy at 2 cm^3^) for all techniques.

For the lung case, the plan with the preset short path is slightly lower in quality than the plan with the body path, and the plan with BOS is slightly lower in quality still, particularly in terms of proximal bronchus and oesophagus dose, but this is a very minor effect. All dose constraints are met, except for the proximal bronchus (18.00 Gy at 4 cm^3^), which is not met by any of the three plans, due to overlap with the PTV.

For the liver case, there is little difference dosimetrically between the three types of plan shown in the DVHs. The PTV constraints (principally 42.75 Gy at 95%) and the normal liver constraints (principally 15 Gy at 50%) are met by all plans. Similarly, with the partial breast case, the techniques are dosimetrically very similar and meet the PTV constraint (35.00 Gy at 95%) and the constraint on the whole ipsilateral breast (17.50 Gy at 40%).

## 4 Discussion

These results show that our fast optimisation scheme is able to produce plans of a clinical standard for predefined beam arrangements such as the body path and the preset short path, within a very practical timeframe. Both the treatment time and the treatment planning time can be reduced significantly by using a preset short path, as both are approximately proportional to the number of beams. The benefit of this type of approach has been shown by a study of standardised beam bouquets for lung planning [36]. The treatment plans in that study contain around six coplanar beams, whereas 36 non-coplanar beams are used in the present study, but the similarity in outcome is clear: carefully chosen standardized beams can produce good quality treatment plans.

However, the greatest benefit in treatment time is expected to be achieved with a BOS path. In this case, as few as 15 beams can be used for the treatment, with plan quality which is almost as high as with the 110-node body path. The BOS path has the shortest treatment time, although as there are similar monitor units and number of apertures for the BOS path compared to the preset short path, the beam-on time and the aperture reshaping times are similar for both paths, and the reduction in treatment time with the BOS path is due to the reduction in robot traversal time. The treatment planning time is much longer with this BOS algorithm, but there are a number of adjustments to the method which would enable it to be used in a much shorter time in a clinical environment, such as limiting the number of iterations for the optimisation at each fixed beam arrangement, and limiting the low-dose extent of the *d_ij_* matrices. Furthermore, it may be possible to use the information gained from this study to design better class solutions without requiring the BOS algorithm to be run for each patient in the clinical environment.

The results of this study are similar to those of Rossi et al. [3] for Cyberknife treatment of prostate with a brachytherapy-like SABR protocol. That study investigates candidate beam sets consisting of a full body path, a coplanar path and three extended body paths consisting of 180–500 nodes. Between 10 and 30 nodes are then selected from these node sets. They find that selecting beams from the largest set of candidate directions favours plan quality. In their study, increasing the number of selected beams from 10 to 30 has little effect on PTV coverage due to the design of the study, but gradually improves the mean dose to the bladder and the irradiated volume of urethra. The impact of increasing the beam number levels off between 15 and 20 beams. The mean dose to the rectum and the rectal irradiated volume also decrease with increasing number of beams, with most of the effect seen with beam numbers up to 20. The finding of the present study that around 15 beams is sufficient to produce good quality plans is in accord with these results. Rossi et al. [3] report optimisation times of up to 45 h, whereas the present work allows an optimisation time of an order of magnitude shorter.

Much of the experience with a C-arm linear accelerator can also be compared with the present study, such as the work of Woudstra et al. [4], Vaitheeswaran et al. [19], Breedveld et al. [8], Amit et al. [20] and Bangert and Unkelbach [23]. Most recently, Liu et al. [21] report on selecting eight beams from either 18 coplanar or 56 non-coplanar candidate orientations for prostate, head and neck, or liver. They find that a sparse optimisation which approximates the exact BOS problem, thereby allowing the use of a gradient method, can provide good quality plans with improved computational efficiency.

To produce such high-quality treatment plans, a series of annular structures around the PTV have been used in the present study. These provide dose distributions evenly balanced around the area treated. The biggest challenge in producing non-coplanar plans with few beams by orientation selection is to ensure that the surface dose is distributed around sufficiently. It has been found that 15 beams are sufficient for this, with 20 beams more than adequate. However, there are some practical limitations to this study. In particular, the method of sequencing the fluence profiles into deliverable segments [34] yields many small segments, with the effect that the total monitor units required are very high. This effect could be overcome by using a more conformal segmentation algorithm. Moreover, no attempt has been made to optimize the monitor units as part of the objective function, but this should be possible. Another limitation is that dose components have been calculated from a small field and the total dose due to a larger field has been calculated as a summation of these elemental doses. It is well known that this is not a very accurate method of calculating the dose in a large field. This could possibly be overcome by applying a segment weight optimisation in a post processing step.

This study begins with candidate beam orientations chosen to avoid collisions. This is similar to the work of Breedveld et al. [8] and Bangert et al. [25]. The present study uses an evolutionary algorithm to select beam orientations, with a fluence optimisation, segmentation and aperture optimisation for each plan at each iteration. However, use of fluence optimisation and aperture optimisation at each iteration may ultimately not be necessary, as fluence optimisation alone may give the required result. As the aperture optimisation problem is posed in this work as a special case of fluence optimisation, so that its speed of execution is approximately the same as the speed of fluence optimisation, removing the aperture optimisation would have the effect of reducing the optimisation time by approximately half. This is also the approach taken by Rowbottom et al. [39] and Hou et al. [38]. Further work is needed to establish what simplifications of the inverse planning can be achieved for the same quality of plan.

## 5 Conclusion

The gradient descent method implemented in a multiple-core computation environment offers the possibility of fast optimisation for MLC-based delivery on the large number of nodes encountered in the Cyberknife system. The number of delivery nodes can be reduced by using a preset short path, but the greatest time saving is achieved by beam orientation selection. The beam selection method takes much longer to run than the standard optimisation method using a fixed set of nodes. However, evolutionary computing produces results which are almost as good in quality as those using the body path. The main advantage of the fewer nodes is expected to be a reduction in treatment time.
